# Firms, activist attacks, and the forward-looking management of reputational risks

**DOI:** 10.1177/14761270221124941

**Published:** 2023-01-12

**Authors:** Estefania Amer, Jean-Philippe Bonardi

**Affiliations:** University of Lausanne, Switzerland

**Keywords:** activism, corporate environmental performance, corporate reputation, private politics

## Abstract

A growing literature investigates how activists launch attacks against firms to improve environmental practices, a situation typically referred to as “private politics.” Whether firms self-regulate in response has been shown to depend on reputational risks. However, reputation management in this literature is mostly reactive, whereas firms could be expected to anticipate and prevent reputation loss when faced with the threat of activism. How they would do so is not obvious, nonetheless, because firms have to consider two opposite effects: (1) a “reputational damage mitigation” effect, through which firms can pre-emptively align to what is expected from them, and (2) a “target enhancing” effect, in which self-regulation makes firms more visible and likely to be criticized. We show, theoretically and empirically, that these two effects actually co-exist and create heterogeneity in firms’ responses when they witness activist attacks in their industry. The real impact of activism on the development of more sustainable practices is thus not only greater than if we solely considered the responses of firms that suffer direct attacks, as many firms start self-regulating before being targeted, but also varies within industries.

## Introduction

The literature on activist attacks against firms has substantially developed in the last two decades (see, for example, Baron and Diermeier, 2007; Eesley and Lenox, 2006; King, 2008; Soule, 2018; Waldron et al., 2013; Zald et al., 2005), looking at how activists pick their corporate targets, how these targets respond, and what happens over time as attacks unfold. Of particular importance for this literature is the impact of these activist attacks on corporate environmental performance (EP). The literature on private politics, which “addresses situations of conflict and their resolution without reliance on the law or government” (Baron, 2003, p. 31),^1^ suggests that the answer to this question depends heavily on the reputational risks involved for firms: When these risks are high, firms tend to address the issues raised by activists; on the other hand, when reputational risks are low, firms have fewer incentives to do so.

This literature, however, tends to focus on reactive responses to attacks (see, for example, Baron and Diermeier, 2007; King, 2008; Lenox and Eesley, 2009; McDonnell and King, 2013), whereas the impact of private politics might depend even more on anticipatory corporate responses to activism: if firms anticipate or try to pre-empt activist attacks with proactive investments in self-regulation, then the effect of these attacks on industry practices should be higher than if this self-regulation is a response that aims at stopping an attack and/or repairing a damaged reputation.^2^ And if activists regularly and instrumentally use reputational damage to attract the public’s attention to questionable practices and to elicit change (Baron and Diermeier, 2007; Breitinger and Bonardi, 2019; Diermeier, 2011; King, 2008; Lyon, 2010), it is reasonable to think that, when facing the threat of activism, managers go beyond reactive approaches and consider the long-term management of their reputation, similar to what happens with other reputational threats (Minor and Morgan, 2011).

In this article, we introduce this forward-looking perspective to reputation management in a theory of how firms handle the threat of activism within their industries, taking into account that, even if activists usually aim to change an industry’s questionable practices by attracting the public’s attention to the latter, limited resources lead activists to target only one or a few industry members at a time (Diermeier, 2011; Soule, 2018; Waldron et al., 2013; Zietsma and Winn, 2008). Given that being selected as a target by a future activist attack can negatively affect a firm’s sales, profits, and stock price returns (Baron, 2003; King and Soule, 2007; Lenox and Eesley, 2009), firms will do their best to avoid future attacks and limit their damaging effects if they think they can. The theoretical implication of this is that firms should try to anticipate reputational risks associated with activism and to develop forward-looking strategies as they attempt to manage their future reputation (Ravasi et al., 2018). These strategies aim not only to avoid the company becoming a target in the future, but also to protect its reputation in case of an activist attack by acquiring reputation insurance (Baron, 2016; Godfrey, 2005; Minor and Morgan, 2011).

Therefore, in this study, we look into (1) how firms respond to the threat of activism within their industry to protect their future reputation, and (2) whether they all respond similarly or there is heterogeneity in their responses. As we will discuss later, answers to these questions are not only informative, helping us to better understand how managers make decisions in difficult situations and manage corporate reputation, but are also critical to evaluating the potential welfare effects of activism, that is, the capacity of activism to encourage firms toward the development of a more sustainable economy.

In the private politics literature, Abito et al.’s (2016) model is a rare exception that looks at reputation in a dynamic and forward-looking perspective. In that context, they acknowledge, for instance, that companies accumulate reputation capital that provides “a cushion in the event of a crisis” (Abito et al., 2016: 244). In the social movements literature, long-term aspects of private politics have also been considered by McDonnell et al. (2015), who explore how some firms that are unresponsive to activism can, when subject to repeated activist attacks, end up becoming responsive.^3^ Apart from these studies, the long-term oriented management of activism-related reputational risks remains surprisingly underexplored in the literature. And yet, it is difficult to grasp the extent to which activism is able to change business practices if we do not properly understand the way companies manage these activism-related risks with a forward-looking perspective. In this article, we develop a theoretical model to better understand this important aspect of private politics.

We hypothesize that managers estimate the threat of activism within their industry based on the attacks that have happened to their company or its industry peers. Therefore, the peer effect is a key element of our approach. The literature shows that companies invest in self-regulation when their industry peers have been the target of shareholder activism (Reid and Toffel, 2009), boycotts (Sam and Innes, 2008), negative press (Durand and Vergne, 2015), and online activism (Zhang and Luo, 2013). Sharkey and Bromley (2015) also find that firms respond to the proportion of industry peers that have been rated for their EP, and that this response is not homogeneous but depends on several factors. In the context of private politics and the firm’s forward-looking strategies to avoid being selected as an activist target and to manage future reputation, the heterogeneity of the peer effect is at the core of our approach.

Predicting how firms will deal with the threat of activism within their industries by using a forward-looking perspective to reputation management is actually not obvious, as at least two contradictory effects are at play. First, once a company perceives an increase in the threat of activism, proactive investments in self-regulation could prevent future attacks and/or mitigate their negative effects on reputation (Baron, 2016; King and McDonnell, 2015). However, another mechanism is that investments in self-regulation can also increase the company’s attractiveness as a target (Luo et al., 2011; King and McDonnell, 2015) and hence the likelihood of being criticized by activists in the future. These two effects, nonetheless, go in opposite directions: the first increases a firm’s incentives to invest in proactive self-regulation, while the second reduces them. For instance, King and McDonnell’s (2015) study finds that firms’ efforts to create and project a socially responsible image makes firms more vulnerable to being targeted by activists and concludes that the second effect is stronger than the first one.

In this article, we contribute to the private politics and social movements literatures by taking a different stance and arguing that, when managers take a forward-looking perspective, the strength of each of these effects varies from one firm to another depending on the characteristics of this firm with respect to its industry peers, which determines the extent to which this firm is subject to the threat of activism. Therefore, managers’ expectations about the threat of activism that a firm is subject to are informed by the overall threat of activism in the industry, and how much this firm is likely to be chosen by activists as a target in the future, which depends on this company’s characteristics with respect to its industry peers. These expectations will determine the extent to which the company is subject to the two aforementioned effects, creating heterogeneity in how firms proactively respond to private politics. This leads us to predict that only firms that are in the middle of their industry’s distribution of EP will address the threat of activism by engaging in proactive investments in self-regulation. We test our model of firms’ responsiveness to the threat of activism using a sample that contains the 350 largest British firms in terms of market capitalization and find empirical support for it.

Thus, our article also contributes to the literature on the heterogeneity of corporate responses to activist attacks. Such heterogeneity has been shown to depend on the extent to which the issue raised by activists resonates with managers and whether they are sympathetic to it (Bundy et al., 2013; Durand et al., 2019; Zald et al., 2005), the company’s culture (Waldron et al., 2013), the managers’ political orientation (Gupta and Briscoe, 2020), the meaningfulness of contested practices (Waldron et al., 2013), whether the company has the resources and capabilities to address the criticism and change its practices (Durand et al., 2019; Zald et al., 2005), or whether managers are able to properly understand the nature of the activists’ concerns and demands (Simmons, 2021). The private politics literature has also modeled the heterogeneity of response to activism by assuming that firms (1) can adopt either “responsive” or “recalcitrant” signaling strategies toward activists (Baron and Diermeier, 2007), (2) are distributed along a continuum of firm vulnerability to activist attacks (Baron, 2016), or (3) can be short-term or long-term oriented (Baron et al., 2016). While all these factors influence firm responsiveness, an important contribution of our study to the literature in private politics and social movements is to show, theoretically and empirically, that the forward-looking orientation to reputation management, which is a long-term oriented approach, is a key determinant of the heterogeneity of firm response to activism.

A second contribution of this article is a deeper insight into the societal impact of the activism-related industry peer effect on firms’ investments in self-regulation. We find that the response to the threat of activism within industries through the peer effect is quite substantial. Therefore, the extent to which activism is able to elicit practice change within industries is more important than if only the direct effect of activism was considered. We believe this is an important result as it enhances the role of activist organizations, and of private politics, in transforming companies and building a more sustainable future.

The rest of the article falls into four sections. The first offers a theory of how firms manage their reputation in a forward-looking manner and develops testable hypotheses. The second presents data and empirical methods, while the third displays the results. The last section discusses these results and concludes.

## Theory and hypotheses

The starting point of our theory is that firms manage their long-term reputation by trying to anticipate the extent to which they are likely to be subject to activism-related reputational risks, and that managers’ expectations about the threat of activism depend on the attacks they have observed in their business environment. In the first subsection, we explain why managers use the industry as a social category of reference to build their expectations about the threat of activism their firm faces. Then, in the second subsection, we focus on how managers actually build these expectations using the information obtained from observing the attacks within the firm’s industry, and how a change in expectations about the threat of activism can lead a firm to proactive investments in more sustainable practices in order to protect its reputation from future potential attacks. Finally, in the third subsection, we theorize about the heterogeneity of responses to an increase in the threat of activism within an industry.

### Strategic decisions on reputational risk management and the industry as the relevant social category of reference

Social categories are defined by external and internal stakeholders in accordance with a number of stable features that these stakeholders see as relevant (Hsu and Hannan, 2005). Organizations belonging to the same social category usually interact with members of each audience in similar ways (Hsu and Hannan, 2005). In the case of companies, those sharing a social category often provide similar goods or services and have similar relationships with their stakeholders (suppliers, clients, regulators, etc.). This also means that they often compete for the same clients or consumers, as well as for the same suppliers. In other words, the members of a social category not only have similar characteristics, but they also share an “ecological niche” (Whetten, 2009: 75) within society.

The industry is often used by external and internal stakeholders as a relevant social category to classify companies and compare them to one another (Hsu and Hannan, 2005; King and Whetten, 2008; Smith and Arnkellson, 2000). Thus, the industry is used in this way not only by the company’s external stakeholders, but also by the company’s managers, employees, and investors. These internal stakeholders also perceive the industry as the social category of reference because industry membership constitutes part of a company’s identity (Hsu and Hannan, 2005; Whetten, 2006). Moreover, the fact that internal stakeholders pay attention to what happens at the level of the industry is a reasonable assumption because companies within an industry are (1) often rivals competing against each other to increase their market share, attract the best suppliers and employees, develop and implement innovations, and so on, and (2) frequently subject to similar pressures from stakeholders, including activists. This makes the industry an invaluable source of information for firms. In sum, what happens in the industry should play a central role as a source of information in managers’ decision processes.

Consistent with this, the management literature shows that companies observe and learn from their industry peers about new markets’ characteristics (Baum et al., 2000; Yue et al., 2013) and the companies’ ability to gain favorable policy outcomes (Bonardi et al., 2006). Sharkey and Bromley (2015) also find that firms that have received an environmental rating consider whether their competitors are also rated or not when they decide whether to reduce their emissions, that is, they integrate what is happening to their competitors in their decision-making processes. Moreover, some industries have responded to reputational crises affecting the whole industry with business-led initiatives and other collective efforts (Barnett and King, 2008; King and Lenox, 2000).

### The assessment of reputational risk within the industry

A central feature of our model is that managers, who carefully oversee their firm’s reputation over time, will not wait for an actual or impending attack to invest in self-regulation, but will rather try to anticipate reputational risks associated with future attacks. In order to learn about these risks, managers use information about activist attacks that have happened within their company’s industry. This is not only because, as mentioned above, the industry is a relevant category of reference, but also because activists, even if they tend to focus their attacks on only one or a few firms at a time, often want to attract public attention to questionable environmental and social practices in this company(ie)’s industry (Diermeier, 2011; Soule, 2018; Waldron et al., 2013; Zietsma and Winn, 2008).

For example, in the 1990s, activist groups that were attempting to eradicate sweatshops and to improve working conditions in the apparel industry’s supply chain targeted Nike because it was an industry leader, even though many other companies in the industry also relied on sweatshops (Den Hond and De Bakker, 2007). Or, in 2019, Greenpeace, to attract public attention to the environmental effects of single-use plastic packaging, which is a questionable practice in the food and beverage industry, sent its activists to demonstrate against this practice at Nestlé’s Annual General Meeting in Lausanne. Activists also sometimes engage in sequential targeting, by moving from one company to the next. For example, in the 2000s, Viva! actively campaigned against the retail industry in the United Kingdom over duck farming practices in the supply chain. But, while demonstrations in 2004 targeted Marks and Spencer, the 2009 Christmas campaign was focused on Sainsbury (Viva!, 2016a, 2016b).

Therefore, in our model, managers use activist attacks that occur in their industry as an important source of information to evaluate the reputational risks associated with future attacks on their own company. To theorize about the way managers perform this risk assessment, we rely on the environmental economics literature on deterrence. This literature shows, both theoretically and empirically, that plant managers use environmental regulators’ past and present inspections and enforcement actions on themselves and their industry peers to estimate the likelihood of future inspections and enforcement actions by these same agencies in the future, which in turn determines these plants’ compliance with environmental regulations (Gray and Shimshack, 2011; Shimshack and Ward, 2005, 2008; Thornton et al., 2005). Similarly, we expect a company’s managers, who have incomplete information about the likelihood that activists will attack their firm in the future, to use the past and present activist attacks within their company’s industry to evaluate the likelihood of a future attack on their company.

If we focus on environmental issues, an activist attack against any given company in an industry should lead the average industry member’s managers to anticipate a rise in the threat of environmental activism, and to an increased awareness of the reputational risks associated with it. If managers adopt a forward-looking orientation to reputation management, this will raise this average industry member’s incentives to proactively invest in forestalling and mitigating environmental self-regulation. The result should be an increase in this firm’s EP.

*H1. An activist attack against a firm in relation to an environmental issue in an industry leads to an increase in the industry members’ EP.*

### Modeling the heterogeneity of response to the threat of activism

In the previous section, we showed that, after an attack within an industry, firms become more aware of the reputational risks associated with potential future activist attacks, raising the average industry member’s incentives to invest in proactive self-regulation. However, not all the companies are equally at risk of becoming activist targets in the future and, as a result, they do not all suffer from the same reputational risks. Since activists pick their targets within the firm’s industry, a manager who wants to assess his or her firm’s reputational risk before making strategic decisions about investments in self-regulation will compare the firm’s attractiveness as a target relative to its industry peers and use this information to determine the ability of proactive investments in environmental self-regulation to reduce the ensuing future reputational risks. If such investments are expected to reduce the future reputational damage associated with activism, managers will decide in favor of these investments. If, on the other hand, they are expected to increase this future damage, they will forego them. We shall see that this depends on the firm’s level of performance in the type of issue that is raised by the activists compared to that of its industry peers, which in this case is related to the natural environment.

**Figure 1.**
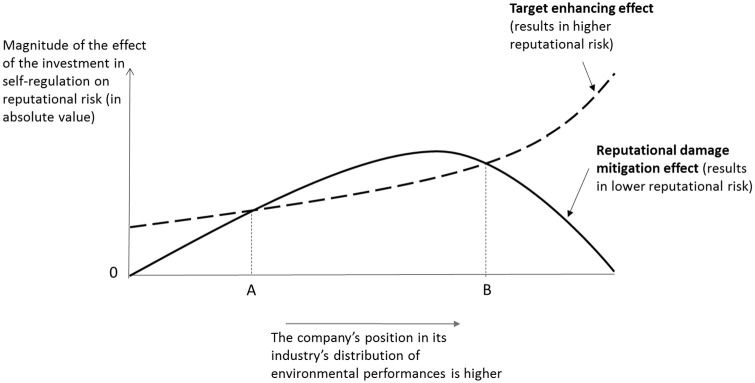
Reputational risks and self-regulation in the context of private politics.

There are, in fact, two effects that should be taken into account and that go in opposite directions. The first is the **reputational damage mitigation effect**, which captures the ability of an investment in proactive self-regulation to reduce the magnitude of future activism-related reputational damage. The second is the **target enhancing effect**, which arises from the fact that, all other things being equal, if a firm invests in proactive environmental self-regulation, it will become a more attractive target and the expected magnitude of future activism-related reputational damage will increase. Both effects are represented in Figure 1, where an industry’s companies are distributed along the horizontal axis according to increasing levels of EP. Environmental laggards within the industry are on the far left of the axis, while the industry’s environmental leaders are on the far right.

The **reputational damage mitigation effect** curve reflects the fact that proactive investments in self-regulation can have two beneficial effects in terms of reducing future activism-related reputational risk, as reported by the literature in private politics (e.g. Baron, 2016; Baron and Diermeier, 2007). First, a firm subject to an increased threat of activism can invest in self-regulation in the hope that activists will appreciate these efforts and be less inclined to target it in the future (forestalling self-regulation). Second, this firm can also use self-regulation to garner public support for the company, mitigating the negative effect of future attacks on its sales and profits (mitigating self-regulation). This mitigating effect has also been explored by the reputation-insurance literature, according to which proactive investments in self-regulation provide a firm with a “reservoir of goodwill” able to protect its future profits from events, such as activist attacks, that could potentially damage the firm’s reputation (Godfrey, 2005; Godfrey et al., 2010; Minor and Morgan, 2011).

Industry members with the lowest levels of EP in Figure 1 are likely to have invested little, relative to their industry peers, in environmental measures. Since reputation-building takes substantial time and effort (Fombrun, 1996; Hall, 1992), the environmental laggards’ first proactive investment in environmental self-regulation after an activist attack is not likely to be credible and convince the public that the company cares about environmental issues. However, as we move up the distribution of EP, we encounter companies that, in the past, have invested more in environmental self-regulation than environmental laggards. These firms, when they make an additional investment in environmental self-regulation, are thus more likely to be credible in their efforts to portray themselves as caring about the environment than the industry’s environmental laggards. Therefore, these firms are more likely to benefit from the forestalling or mitigating effects of self-regulation than the companies at the bottom of the industry’s distribution of EP. In sum, we expect the **reputational damage mitigation effect**, which reduces the future reputational risk, to increase in magnitude as we move from the bottom and upward in the industry’s distribution of EP.

However, once we reach a certain level of EP within the industry’s distribution, we expect the magnitude of this mitigating effect to start to decrease. Indeed, the positive impact of corporate social responsibility (CSR) investments on a company’s reputation seems to have an upper limit (Wei et al., 2014), which means that at some point additional investments in CSR fail to provide additional reputation insurance. Similarly, Minor (2011) found that being a “stellar” corporate citizen by engaging in CSR expenses beyond what responsible companies already spend does not seem to provide additional reputation insurance benefits. The existence of this upper limit is also why good environmental performers within an industry have incentives to “coast” on their reputation (Abito et al., 2016). Therefore, we expect the **reputational damage mitigation effect** curve to be concave. While in Figure 1 we represent the effect as becoming null only when we reach the top performer, it is possible that it is actually so for more than one company at the top. However, even if this was the case, the main conclusion of the model would remain unchanged.

The **target enhancing effect**, on the other hand, captures the fact that investments in proactive environmental self-regulation can make the company more attractive to environmental activists. The latter regularly use the media to make environmental and social issues salient (Bonardi and Keim, 2005), and public support is usually crucial for the success of activist campaigns (Baron, 2016; Baron and Diermeier, 2007). Therefore, activists have a strong incentive to choose companies that will maximize the likelihood that the attack will be picked up by the media. A good environmental performer that has nevertheless engaged in environmentally questionable practices can easily become the villain of a dramatic news story about hypocrisy because the media know that this kind of reporting is particularly appealing to the public (Diermeier, 2011). Moreover, from an activist’s perspective, when a company that has acquired a reputation for being a good environmental citizen, and has thus created expectations among stakeholders, is subsequently caught engaging in environmentally questionable practices, it can be perceived as being hypocritical and spark public outrage, putting a target on the company’s back (King and McDonnell, 2015).

Something similar happens with social practices. For example, in 1999, Global Exchange, a non-governmental organization (NGO) focused on human rights that wanted to pressure the coffee industry to adopt fair trade practices, targeted Starbucks, one of the CSR leaders in its industry. In 2002, Deborah James, Global Exchange’s Fair Trade Director, reported that they focused on Starbucks “because we knew that they put themselves in the public eye as a socially responsible company. The fact that Starbucks is using that as part of their marketing and advertising leaves them open to being exposed when they are not socially responsible. So the fact that they say that they are socially responsible opens the door for us to target them” (Gallander, 2002). It should be noted that, while a company can end up being perceived as an environmental leader because it has publicized its good deeds, it can also become an environmental leader in the public’s eyes simply because the media or other actors have reported about its environmental record and/or through the Environmental, Social, and Governance (ESG) ratings published by some agencies and organizations.

To sum up, targeting a good environmental performer within the industry raises the likelihood that the activist attack, which aims at drawing attention to the industry’s questionable environmental practices, will be picked up by the media. As a result, activists wanting to attract public attention to an industry’s questionable environmental practice have strong incentives to go after environmental leaders. If a firm that is already high in the industry’s distribution of EPs made additional investments in environmental self-regulation, it would probably go up in the industry’s distribution of EPs and attract even more public and activist attention to itself, and the company’s managers know this. This is why the managers of a company that is high in its industry’s distribution of EP have few incentives to invest in environmental self-regulation, even if these managers have witnessed an attack within the industry and, as a result, raise their expectations regarding the threat of environmental activism. In 2000, as Starbucks was under attack by Global Exchange, the firm’s managers were aware that succumbing to this activist’s demands could signal to other activists that Starbucks was an “easy target” for future campaigns (Argenti, 2004: 104).

On the other hand, a company that is relatively low in the industry’s distribution of EPs can also attract attention if it invests in environmental self-regulation, but to a lesser extent, as environmental leaders remain very attractive targets. Therefore, we hypothesize that the magnitude of the **target enhancing effect** increases as we move up the distribution, and is particularly strong for the best environmental performers in the industry, which explains the form of the curve (Figure 1).

Since, in Figure 1, the **reputational damage mitigation effect** and the **target enhancing effect** capture, respectively, the magnitudes of the reduction and of the increase of activism-related reputational risk resulting from a given proactive investment in environmental self-regulation, only industry members for which the first curve is above the second one have incentives to respond to an activist attack with such an investment. These members are companies in the middle of their industry’s distribution of EPs (between points A and B). The companies at the bottom of the distribution will not invest in proactive self-regulation after an attack within the industry because they have very little to gain in terms of forestalling or mitigating self-regulation. For these companies, building a credible reputation for caring about the environment would require time and substantial efforts in terms of investments. As long as they remain at the bottom of their industry’s distribution of EPs, any proactive investment in environmental self-regulation would have a negligible mitigating effect on reputational risk. The firms at the top of the distribution also have few incentives to carry out such investments after an activist attack within the industry because the **target enhancing effect** offsets the forestalling and mitigating benefits of additional investments in environmental self-regulation.

*H2. An activist attack against a firm’s industry peer in relation to an environmental issue leads to an increase in this firm’s EP, but only if this firm is in the middle of its industry’s distribution of EPs, and not if it is at the distribution’s extremes.*

## Data and methodology

The sample contains the constituents of the FTSE 350 stockmarket index on 31 December 2012, that is, the 350 largest companies listed on the London Stock Exchange in terms of market capitalization. We focus on British companies because the source of the data concerning the activist attacks, the Corporate Critic Database (CCD), is a product of the Ethical Consumer Research Association (ECRA), a British not-for-profit consumer movement that has been “challenging corporate power since 1989” (ECRA, 2016).

### Dependent variable

The indicator of EP on the last day of each year between 2001 and 2011 was retrieved from the Asset4 database (Thomson Reuters). Its values are between 0 and 100. The Asset4 scores have already been used in other CSR-related empirical studies (Barnett et al., 2018; Cheng et al., 2014; Luo et al., 2015; Zyglidopoulos et al., 2012). According to Barnett et al. (2018), the Asset4 environmental score is the most comprehensive indicator of EP available.

It should be noted that while Thomson Reuters uses the media as a source of information to calculate the Asset4 environmental score, our explanatory variable also relies on that source. However, this is not a problem in this study. First, the news media is only one of many sources used by Thomson Reuters to calculate their environmental score. Moreover, endogeneity sources that lead to biased coefficients should be captured by the Sargan test, which would reject the null hypothesis that the overidentifying restrictions are valid. Our results will show that this is not the case.

### Independent variables

Our independent variable is *Peers activist attack*, which captures whether at least a firm’s industry peer has suffered one or more activist attacks in relation to an environmental issue in a given year. It is thus a proxy of the activist pressure related to this type of issue on this firm’s industry in that year. To capture whether an activist attack is able to elicit a forward-looking response to the threat of activism in order to protect a firm’s reputation in the future, we do not include attacks on that firm (the focal firm), because this firm’s response could simply aim at stopping the attack or repairing the company’s reputation immediately after an attack.

To calculate *Peers activist attack*, we used the 1995–2012 data available in the CCD, provided by the ECRA, a consumer association that engages in activism. The ECRA collects data on environmental and social issues related to companies from individual activists’ and civil society organizations’ publications and websites, as well as from major British and non-British media outlets, including, but not limited to, *BBC News*, *Financial Times*, *The Daily Telegraph*, *The Guardian*, *The Independent*, *The New York Times*, *The Observer*, *The Times*, *The Wall Street Journal*, and *The Washington Post*. ECRA also retrieves information on the companies’ environmental and social records from other public sources, including governmental agencies and corporate communications. With all this information, the ECRA generates company records for each event related to a socially or environmentally related issue, which it uses to calculate each company’s ethical rating (Ethiscore). ECRA also launches boycott calls against companies or directly reports about the companies’ environmental and social records in its *Ethical Consumer Magazine*.

These records, available in the CCD, allowed us to first construct *Peers activist attack_it_*, which is a dummy variable that is equal to unity if at least one of company *i*’s industry peers has been attacked by an activist in relation to an environmental issue at least once during year *t*, and zero otherwise. We relied on two additional versions of *Peers activist attack*, to make sure that we captured the peer effect adequately. The second version of *Peers activist attack* is the number of industry peers that have been attacked in such a way at least once in a given year. Finally, we used a third version of *Peers activist attack*, the natural logarithm of this number of industry peers plus one, because the marginal effect of the number of peers attacked in a given year on the company’s response may be decreasing as the number of peers attacked increases. While the first version captures whether there is a difference between no attacks on industry peers and at least one, the two other versions of *Peers activist attack*, which rely on the number of peers attacked, allow capturing how companies respond to the overall pressure exerted on their industrial sector by activism in a given year.

Only the CCD records where there was at least one activist attack on a company in relation to an environmental issue were used to calculate the independent variables’ values for this company. They primarily include information about boycott calls and campaign launches, as well as criticism in reports, websites, and newsletters from NGOs (*Greenpeace*, *Friends of the Earth*, *PETA*, etc.), as well as in other non-profit organizations’ or activist-journalists’ magazines and reports that promote the green agenda (e.g. *Earth Matters*, *Animal Times*, *Vegetarian*, *Down to Earth*, *Ethical Consumer Magazine*).

The first author carefully read all the CCD records (or entries) and assigned them to two categories: negative and positive/neutral (a document with the detailed procedure followed to collect data from the CCD is available upon request). Only negative records were used to compute the independent variables. A second rater independently coded the same records using the same two categories mentioned above. Interrater reliability was good, with a Cohen’s kappa value of 0.90 (Cohen, 1960), suggesting a high level of consistency across the two raters.

To determine which companies are industry peers, we used each company’s two-digit SIC code as reported by Worldscope (Thomson Reuters). A peer is any company in our sample of British companies that has the same two-digit SIC code as the company of interest, an identification strategy that has already been used by Reid and Toffel (2009). Peers are also often defined as plants or companies that are not only in the same industry, but also in the same geographic area (Gray and Shadbegian, 2007; Shimshack and Ward, 2008). In our study, we consider that the United Kingdom is small enough for all companies within an industry to be likely to learn about an activist attack on a British industry peer.

### Company-specific time-varying control variables

Our identification strategy includes company-specific fixed effects. While these allow controlling for company-specific time-invariant characteristics, they do not control for time-varying ones. Hence, we introduce firm-level time-varying control variables that could potentially bias the coefficient estimates in all our regression models.

As mentioned in the previous subsection, an attack on a focal firm is a potential confounding factor in our identification strategy, because if an activist attacked the focal firm and one of its peers simultaneously, and we observed a response, it could be due to the focal firm’s effort to repair its reputation, rather than the forward-looking response we have hypothesized. Moreover, the effect of the attacks suffered by peers on a company’s decisions regarding its environmental practices could depend on the direct activist attacks that this company has suffered in previous years.

To control for this potential source of endogeneity, we constructed *Focal firm activist attack_it_*, a dummy variable that is equal to unity if company *i* has been attacked by an activist in relation to an environmental issue at least once during year *t*, and zero otherwise, using the CCD database. In sum, while we rely on *Peers activist attack* to test our hypotheses, we control for direct attacks on firms with the control variable *Focal firm activist attack.*

Company size can increase the likelihood of activist attacks, but it can also provide the company with more resources either to invest in environmental measures or to fight back against activist attacks and repair its damaged reputation, reducing the likelihood of being targeted (Eesley and Lenox, 2006; Ioannou and Serafeim, 2012; Lenox and Eesley, 2009). Thus, we introduced a company’s *Assets* and *Net sales* to control for company size. An easier access to liquid capital also facilitates investments in environmental measures and pushing back against activists (Adams and Hardwick, 1998; Eesley and Lenox, 2006; King, 2008; Lenox and Eesley, 2009). Therefore, we introduced *Cashflow*, a proxy of the financial resources available, which we calculated using the same procedure as Eesley and Lenox (2006), and the company’s stock of *Cash.* Moreover, profitable companies are more likely to have financial resources they can dedicate to environmental investments (Adams and Hardwick, 1998) and they may also be more publicly visible and thus more likely to be targeted by activists. Therefore, the annual return on assets was introduced to control for company *Profitability*. Because high levels of debt may render a company more visible in the media and reduce the resources available for environmental investments (Adams and Hardwick, 1998), we have also controlled for a company’s *Leverage*. This variable is equal to the company’s debt as a percentage of total assets. We retrieved all the data required for these variables from the Worldscope database (Thomson Reuters).

Finally, we also introduced *Media visibility*, a proxy for a company’s public visibility, as an additional control variable. *Media visibility* is equal to the number of times a company has appeared in the major English-language press publications each year, regardless of the issue discussed and the tone of the news (positive, negative, or neutral). They were retrieved from the LexisNexis database selecting the option “exclude share indexes.”

### Identification strategy

To evaluate whether an activist attack on a company in relation to an environmental issue is a driver of a company’s increase in EP, we use the following model

$$EP_{it}=\alpha +\overset{J}{\underset{j=1}{\sum }}\theta_{j}EP_{i,t-j}+\overset{K}{\underset{k=1}{\sum }}\beta_{k}Peers\;activist\;attack_{i,t-k}+\overset{K}{\underset{k=1}{\sum }}\delta_{k}X_{i,t-k}+\mu_{i}+\tau_{t}+\varepsilon_{it}$$

where *EP_it_* is company *i*’s EP in year *t*, *Peers activist attack_it_* captures whether at least one of company *i*’s peers has been attacked by an activist concerning an environmental issue at least once in year *t* and zero otherwise. *J* and *K* are the number of lags of *EP* and *Peers activist attack* included in the model, respectively. *X_i, t−k_* is a column vector that contains the lagged company-specific time-varying control variables.

Company-fixed effects (*µ_i_*) and time-fixed effects (*τ_t_*) were also introduced in the model. The model also contains lags of the dependent variable on the right-hand side (RHS) of the equation, resulting in a dynamic panel data model. These lags were introduced because a company’s present EP, past activist attacks, which are captured in *Industry activist attack*, and past profitability can depend on this company’s previous EP. For example, a company that has an Environmental Management System (EMS) in place in a given year is also more likely to have one in the following year. Moreover, the presence of lags of the dependent variable on the RHS of the equation controls for any endogeneity arising from reverse causality (Leszczensky and Wolbring, 2022) and, more generally, from any estimation bias resulting from the correlation of past values of EP with its present value and any of the RHS variables.

The coefficient of interest, *β_k_*, should be interpreted as how much higher a company’s EP at time *t* is, on average, when there has been at least one peer that has suffered one or more activist attacks within the industry regarding an environmental issue *k* years before (i.e. in year *t−k*), with respect to the EP it would have if no peer attacks had happened in year *t−k*.

Since the fixed-effects estimator yields inconsistent coefficient estimates for dynamic panel data models (Nickell, 1981), we had to use the Arellano–Bond estimator, which is an IV-GMM procedure that is applied to the first-differences transformation of the regression model. Contrary to the fixed-effects estimator, the latter provides unbiased estimates for dynamic panel data models (Cameron and Trivedi, 2005). This estimator has already been used in the management literature when the presence of lags of the dependent variable on the RHS of the equation precluded other estimation procedures (Alessandri et al., 2012; Bapna et al., 2013). All our regression models, which rely on the “classical” one-step Arellano–Bond difference GMM estimator (Arellano and Bond, 1991), were run using STATA 13’s *xtabond* command. Finally, an advantage of the Arellano–Bond estimation procedure is that it relies on an overidentified instrumental variable procedure. This procedure yields consistent (or unbiased) estimates of the coefficients as long as the assumption that the instruments are truly exogenous is valid, and the overidentification allows testing this exogeneity hypothesis with the Sargan test.

## Results

Table 1 reports the descriptive statistics and the pairwise correlations among the variables. If only the subsample of companies for which there is a full set of data is considered, the means of the variables do not significantly differ from the means of the whole sample.

**Table 1.** Descriptive statistics of the variables and pairwise correlations.

Variable	Number of obs.	Mean	SD	Min	Max	(1)	(2)	(3)	(4)	(5)	(6)	(7)	(8)	(9)	(10)	(11)	(12)
(1) Environmental performance	2147	61.56	26.91	9.59	97.18	1.000											
(2) Peers activist attack (dummy)	6300	0.34	0.47	0.00	1.00	0.067	1.000										
(3) Peers activist attack (number of peers attacked)	6300	0.63	1.11	0.00	6.00	0.082	0.793	1.000									
(4) Peers activist attack (number of peers attacked, natural logarithm)^a^	6300	0.33	0.51	0.00	1.95	0.084	0.916	0.967	1.000								
(5) Focal firm activist attack	6300	0.07	0.26	0.00	1.00	0.238	0.212	0.222	0.236	1.000							
(6) Assets	5106	17.72	111.52	0.00	2394.57	0.184	0.129	0.167	0.169	0.249	1.000						
(7) Net sales	5077	4.00	14.83	0.00	293.30	0.259	0.079	0.075	0.083	0.355	0.379	1.000					
(8) Cashflow	4922	0.42	1.71	−20.39	26.49	0.246	0.087	0.096	0.100	0.314	0.235	0.828	1.000				
(9) Cash	4782	0.27	1.01	0.00	20.96	0.193	0.004	0.010	0.009	0.110	0.723	0.473	0.362	1.000			
(10) Profitability	4889	7.35	11.72	−127.76	185.33	−0.146	−0.014	−0.004	−0.007	0.004	−0.070	−0.022	0.077	−0.043	1.000		
(11) Leverage	5054	21.16	19.78	0.00	206.38	0.092	−0.008	0.013	0.006	0.032	−0.001	−0.017	0.000	−0.051	0.027	1.000	
(12) Media visibility	6300	231.64	1348.43	0.00	60,631	0.108	0.055	0.063	0.066	0.186	0.225	0.212	0.190	0.102	0.009	0.036	1.000

SD: standard deviation.

a Before calculating the natural logarithm we add one to the number of peers, so that when no peer is exposed in one given year, the value of this variable is equal to zero.

Table 2’s Columns (1) to (4) report the coefficient estimates of the full model as described in the “Identification strategy” subsection. Columns (1) and (2) show the fixed-effects and Arellano–Bond coefficient estimates, respectively, using the dummy version of peers activist attack, while in Column (3) the value of this variable is equal to the number of industry peers that have been attacked in a given year, and in Column (4) this variable is equal to the natural logarithm of this number plus one.

**Table 2.** Effect of activist attacks in relation to environmental issues on the company’s environmental performance.

Variables	Three lags of each control variable (80 instruments)				One lag of each control variable (64 instruments)		
	(1) Fixed-effects	(2) Arellano–Bond	(3) Arellano–Bond	(4) Arellano–Bond	(5) Arellano–Bond	(6) Arellano–Bond	(7) Arellano–Bond
Environmental Performance at *t*−1	0.19*** (0.037)	0.38*** (0.059)	0.37*** (0.059)	0.37*** (0.059)	0.35*** (0.053)	0.35*** (0.053)	0.35*** (0.053)
Environmental Performance at *t*−2	0.03 (0.03)	0.14*** (0.035)	0.14*** (0.034)	0.14*** (0.035)	0.11*** (0.035)	0.11*** (0.034)	0.11*** (0.035)
Peers activist attack at *t*−1 (*dummy*)	−0.29 (0.99)	−0.21 (1.47)			0.014 (1.42)		
Peers activist attack at *t*−2 (*dummy*)	0.67 (1.17)	1.52 (1.26)			2.10* (1.25)		
Peers activist attack at *t*−3 (*dummy*)	−0.66 (1.11)	0.25 (1.34)			0.68 (1.32)		
Peers activist attack at *t*−1 (*number of peers attacked*)			−0.044 (0.65)			−0.072 (0.65)	
Peers activist attack at *t*−2 (*number of peers attacked*)			1.73** (0.71)			1.74** (0.74)	
Peers activist attack at *t*−3 (*number of peers attacked*)			0.94 (0.63)			0.86 (0.62)	
Peers activist attack at *t*−1 (*number of peers attacked, natural logarithm*)				−0.28 (1.48)			−0.21 (1.45)
Peers activist attack at *t*−2 (*number of peers attacked, natural logarithm*)				3.13** (1.50)			3.42** (1.52)
Peers activist attack at *t*−3 (*number of peers attacked, natural logarithm*)				1.36 (1.46)			1.42 (1.44)
Company-fixed effects	Included	Included	Included	Included	Included	Included	Included
Time-fixed effects	Included	Included	Included	Included	Included	Included	Included
Observations	1419	1170	1170	1170	1232	1232	1232
*R* ^2^	0.183						
Number of companies	245	232	232	232	236	236	236
Number of instruments		80	80	80	64	64	64
Sargan test *p* value		0.254	0.325	0.310	0.282	0.362	0.344
Arellano–Bond test *p* value							
Order 1		0.000	0.000	0.000	0.000	0.000	0.000
Order 2		0.844	0.760	0.768	0.728	0.697	0.686

The dependent variable is *Environmental Performance at t.* The unit of observation is the company. The estimator used (either fixed-effects or Arellano–Bond) is specified at the top of each column. Time- and company-fixed effects are included in all the models. The Sargan test null hypothesis is that the overidentifying restrictions are valid. The Arellano–Bond test null hypothesis is that there is no autocorrelation of the first-differenced error terms. Below each coefficient, robust standard errors of coefficient estimates are reported in parentheses.

* *p* < 0.10; **p < 0.05; ***p < 0.01.

The comparison of the coefficient estimates of the lags of EP of Table 2’s Columns (1) and (2) shows that the fixed-effects estimate of the coefficient for the lagged dependent variable seems to be downward biased, as predicted by econometric theory (Nickell, 1981). Therefore, to obtain consistent coefficient estimates, we had to rely on the Arellano–Bond estimator for all the other models in this study. The instruments used by this estimator are the lags of the dependent variable and the first differences of all the independent and control variables as instruments. In Columns (1) to (4), the instruments include the second and all subsequent lags of the dependent variable, resulting in an instrument count (or number of instruments) of 80.

Columns (2), (3), and (4) use the three versions of *Peers activist attack*: the dummy, the number of peers attacked, and the natural logarithm of this number, respectively. While the second lag of *Peers activist attack* is positive and significantly different from zero in Columns (3) and (4) with *p* values of 0.015 and 0.037, in Column (2), the *p* value is 0.225, but the coefficient is still positive. These results show that a company responds to an activist attack on one or more industry peers with an increase in EP, but that this response only happens 2 years after the attack. This is consistent with Hypothesis 1, according to which an activist attack on an industry leads to an increase in the industry members’ EP through the forward-looking management of the reputational risk associated with potential future activist attacks.^4^

Table 2’s results are robust to (1) the addition of an extra lag of the dependent variable or of each of the independent variables on the RHS of the regression model, and (2) replacing the levels of the control variables *Assets*, *Net sales*, *Cashflow*, *Cash*, and *Media visibility* with their natural logarithms. Moreover, the Sargan test *p* values in Table 2 do not allow us to reject the null hypothesis that the Arellano–Bond instruments are truly exogenous, which alleviates potential concerns that omitted variables could bias the coefficient estimates. Finally, Table 2’s *p*-values of the Arellano–Bond test also indicate that there is a first-order autocorrelation between the first-differenced error terms, which is expected by construction, but no second-order autocorrelation that could compromise the validity of the results (Roodman, 2009b).

According to Roodman (2009a), the Arellano–Bond estimator can be affected by a problem of proliferation of instruments (i.e. too many instruments), which can occur when the instrument count is close to the number of individuals. In Table 2’s Columns (2), (3), and (4), the estimation procedure relies on 80 instruments, which is due, at least in part, to the high number of control variables. Indeed, the Arellano–Bond estimation procedure uses one or more lags of the dependent variable and the first differences of all the explanatory and control variables as instruments (Cameron and Trivedi, 2005, p. 765). In these three columns, while the number of instruments is 80, the sample size is 232. Therefore, a problem of proliferation of instruments is unlikely. However, we decided to run regression models of Columns (2)–(4) using only one lag of each control variable, which allows lowering the number of instruments from 80 to 64, and we report the results in Table 2’s Columns (6)–(8), respectively. The results are robust to this change.

Moreover, it should be noted that our 64-instrument specification uses the second and all the subsequent lags of the dependent variable. While the first differences of all the explanatory and control variables must be used as instruments, it is possible to further reduce the instrument count by using fewer lags of the dependent variable. By doing so, we further lowered the instrument count (54 instead of 64) and, once again, our results were robust to this change. These results show that a problem of proliferation of instruments in Table 2’s models is highly unlikely.

To test Hypothesis 2, we first ordered companies within each industry according to their intertemporal median of *EP* by using the two-digit SIC code. Then, within each industry, we split the companies into three same-size groups, that is, three tertiles. We group all the companies in the bottom tertile of their industry in one single subsample, called “Bottom Tertile.” We do the same for the “Middle Tertile” and the “Top Tertile.” We ran our benchmark model, where *Peers activist attack* is defined as a dummy variable, using the 54-instrument specification mentioned in the previous paragraph. These results are reported in Table 3. Even if all results obtained with the 54-instrument specification are also robust to using the 64-instrument set, we prefer to use the former instead of the latter because a split-sample approach reduces the sample size, and with a lower sample size the risk of a problem of proliferation of instruments increases.

**Table 3.** How a company’s environmental performance with respect to its industry peers influences its response to activist attacks on industry peers.

Variables	(1)	(2)	(3)	(4)
	Environmental performance within each industrial sector			
	Bottom tertile	Middle tertile	Top tertile	*Robustness check with extremes of distribution* (*top* *+* *bottom tertile*)
Environmental Performance at *t*−1	0.33*** (0.086)	0.27*** (0.074)	0.36*** (0.071)	0.49*** (0.08)
Environmental Performance at *t*−2	−0.08 (0.08)	0.15*** (0.05)	0.095** (0.05)	0.10** (0.05)
Peers activist attack at *t*−1 (*dummy*)	−0.83 (2.19)	1.85 (2.59)	−0.87 (1.91)	−1.34 (1.56)
Peers activist attack at *t*−2 (*dummy*)	1.04 (2.24)	5.80*** (1.97)	−0.05 (1.90)	−0.05 (1.56)
Peers activist attack at *t*−3 (*dummy*)	4.06 (2.59)	1.89 (2.10)	−1.58 (1.75)	−0.19 (1.59)
Control variables at *t*−1	Included	Included	Included	Included
Company-fixed effects	Included	Included	Included	Included
Time-fixed effects	Included	Included	Included	Included
Observations	225	452	555	780
Number of companies	50	86	100	150
Number of instruments	53	54	54	54
Sargan test *p* value	0.150	0.253	0.190	0.924
Arellano–Bond test *p* value				
Order 1	0.000	0.000	0.000	0.000
Order 2	0.727	0.468	0.095	0.302

The dependent variable is *Environmental Performance at t.* The unit of observation is the company. The Arellano–Bond estimator is used in all the regression models. The Sargan test null hypothesis is that the overidentifying restrictions are valid. The Arellano–Bond test null hypothesis is that there is no autocorrelation of the first-differenced error terms. Below each coefficient, robust standard errors of coefficient estimates are reported in parentheses.

* *p* < 0.10; **p < 0.05; ***p < 0.01.

Table 3’s Columns (1) to (3) estimates of *Peers activist attack* show that only the companies in the middle of the distribution respond to the attacks in relation to environmental issues that their peers have suffered. For the middle-of-the-distribution subsample, the second lag’s coefficient estimate of *Peers activist attack* is 5.8 and its *p* value is 0.003, with a 95% confidence interval of (1.9, 9.7). Given that the EP indicator’s value is between 0 and 100, if a single attack on a company is able to elicit such an effect on other industry members, and even if it only affects one-third of them, our results suggest that the mere threat of activism plays an important role in changing environmental practices within an industry through the peer effect.

At the same time, our results show that companies in the “Bottom Tertile” and “Top Tertile” subsamples do not respond to activist attacks on their industry peers. However, there is a concern with the regression results for the “Bottom Tertile” subsample: the instrument count, which is 53, exceeds the sample size (50 companies). To address this issue, and in order to confirm the absence of the peer effected in the extremes of the industries’ distribution of EPs, we merged the “Bottom Tertile” and “Top Tertile” subsamples into a single one and reported the results in Table 3’s Column (4). With a sample size almost three times the instrument count, we can confirm that our data support Hypothesis 2. While we cannot exclude that the peer effect may exist for some companies at the top or the bottom of the distribution of EPs within their industries, the effect hypothesized is only observed for companies in the middle of the distribution.

A key element of our model is our assumption that the **target enhancing effect** increases as the company’s EP with respect to its industry peers goes up. The data clearly support this assumption. During the period considered, companies in the “Top Tertile” and “Middle Tertile” were, on average, and, respectively, 4.1 and 2.2 times more likely to be attacked than companies in the “Bottom Tertile.” This also means that companies in the “Top Tertile” subsample were the most frequently attacked and yet they do not respond to attacks within their industries, as predicted by our model.

## Discussion

In this article, we use insights from the literatures on private politics, corporate reputation, social movements, and sustainability to develop a theoretical model of companies’ responsiveness to the threat of activism within their industries by considering long-term management of reputational risks. Our hypotheses predict that investments in proactive environmental self-regulation take place: (1) when a firm witnesses an activist attack within its industry, and (2) only for companies in the middle of the distribution of their industry’s EP.

Empirically, we find that the bulk of the increase in the company’s EP happens 2 years after the attack, which is consistent with the idea that these investments are not “quick fixes” made during or just after the attack, such as environmental philanthropy, but aim at protecting the firm’s reputation in the longer term. An alternative explanation for the peer effect could be that, after observing the implementation of environmental measures in the attacked company, industry peers implement similar measures so as not to be left behind (Guler et al., 2002). However, in such a case, we would observe an increase in EP first in the targeted company and only later in its industry peers, which is not the case here. While imitation and the diffusion of environmentally friendly practices within an industry happen independently of activism, our results are consistent with the forward-looking mechanism we have hypothesized.

According to our model, whether an industry member has incentives to invest in self-regulation depends on the interplay of two effects resulting from investments in self-regulation: The **reputational damage mitigation effect**, which reduces future activism-related reputational risk, and the **target enhancing effect**, which increases it. Our model predicts, and our results show, that only companies in the middle of the industry’s distribution of EPs respond to activist attacks within their industry in relation to environmental issues with an increase in EP. And yet our results show that the top environmental performers, despite a lack of responsiveness, are the most frequently attacked in their industry. Since these top environmental performers are particularly visible to the public and their industry peers as far as environmental issues are concerned, activists’ targeting choices are usually directed at them. Indeed, even if our data suggest that an attack on a good environmental performer does not elicit changes in the targeted company, it increases the likelihood that the attack is picked up by the media, which would positively contribute to industry-wide change through the peer effect.

### Contributions and implications

We believe our article makes several contributions, in particular to the literature on how firms manage their reputation when facing crisis situations, to the CSR literature, and more broadly to the sustainability literature, especially regarding the role private politics must play in order to build a more sustainable economy.

First, a key implication of our study is that the management of reputation is a forward-looking phenomenon, and is less static or reactive than suggested in some core models of private politics (see, for example, Baron and Diermeier, 2007). In doing so, we emphasize the importance of peer effects in activism-induced practice changes, which have been highlighted by some seminal papers (Reid and Toffel, 2009; Sam and Innes, 2008; Zhang and Luo, 2013). In fact, the peer effect appears to be very strong in our results, and it seems to be able to elicit important changes in the companies’ EP. Here, we have considered this forward-looking managerial stance mostly from the perspective of the private politics literature, but it would be interesting to further develop this perspective in the broader reputation management literature. Reputation management can be considered from a negative perspective, as we do here—what might cause a reputation damage (Breitinger and Bonardi, 2019)—but also from a positive one—how good reputations can be built (Rindova, 1997). Both should include forward-looking perspectives, as good reputations could be built both from reactions to events but also from planned and anticipated activities developed over time (Minor and Morgan, 2011), which create consistency (Roberts and Dowling, 2002). It would be interesting to see this point considered more specifically in future studies.

Going back to the literature on firm–activist relationships, and beyond the forward-looking perspective on the management of reputation, our article makes a contribution to our understanding of the heterogeneity in firms’ responses to activist challenges. This heterogeneity has already been considered in the private politics literature, but it has been considered as mostly exogenous. In Baron and Diermeier’s canonical piece, for instance, firms are assumed to be ex ante of two different kinds: the soft ones, which will back down and respond positively to activist criticism, and the recalcitrant ones, which will fight and refuse to adjust their practices (Baron and Diermeier, 2007). We show in this article that this dichotomy could actually be the product of a calculation by managers, depending on their firm’s relative position in the industry in terms of EP. From that perspective, it becomes an endogenous choice for firms made in anticipation of future activist attacks.

In this regard, another contribution of this article, both to the private politics and the CSR literatures, is to consider jointly the **target enhancing effect** and the **reputational damage mitigation effect** in order to derive a model that explains the heterogeneity of responses to the threat of activism within an industry based on a forward-looking perspective to reputation management. These two effects have been highlighted by two different streams in the literature, leaving the field overall with a puzzle: Firms can protect themselves from activist attacks by investing in environmentally responsible measures, which provides them with reputation insurance (Godfrey, 2005; Minor and Morgan, 2011), but might also refrain from doing so because they fear becoming too visible and thus an ideal target for future attacks (Graafland, 2018; King and McDonnell, 2015). So, a legitimate question remains: Which of the two effects dominates? We show in this article that these two effects actually exist and co-exist, and that which one dominates the other depends on the EP of the firm considered with respect to its industry peers. For some, the **target enhancing effect** dominates, while, for others, it is the **reputational damage mitigation effect** that does. We thus reconcile these two streams of literature and show that they are not mutually exclusive when one takes into account a forward-looking perspective of how firms manage reputational risks and investments in environmentally and socially responsible measures.

Interestingly, and even though we do not focus on this here, our article has implications for another strand of the private politics literature: namely, the one that concentrates on activists’ targeting strategies (Breitinger and Bonardi, 2016; Eesley et al., 2016). Indeed, the private politics literature has two subparts: (1) the targeting literature—why certain firms are targeted rather than others, and (2) the firm-response literature—how firms respond to activists’ attacks. Our article mostly contributes to the latter, but it has implications for the former. If the firms that respond are mostly the ones that are in the middle of the distribution regarding EP, one would expect that those firms are also the ones that get criticized the most. The problem with this argument, however, is that activists might want to go after certain firms not because they will be quick to adjust their own environmental practices, but rather because they will lead many others to pre-empt attacks and invest in more sustainable activities. This argument is in line with Rehbein et al. (2004) who find that activists target larger, more visible, and more consumer-oriented firms. Here again, the industry peer effect is critical, and it would be interesting to revisit the targeting literature from that angle and explore how activist organizations adapt their tactics accordingly (Eesley et al., 2016; Lyon, 2010).

Our study also shows the important role of the industry, as a category of reference, in activist–company relationships. While most activists strategically select their targets within the industry with the objective of achieving maximum impact and change within it, companies in our model use the industry as a source of information and an important category of reference to respond to activist attacks. In these industry-level activist–company interactions, company reputation has a central role, as both activists and companies are constantly trying to shape the latter’s reputations (Ravasi et al., 2018).

Finally, our article could be seen from a broader “welfare” perspective, that is, from the perspective of the necessary changes that need to occur in business practices in order to build a more sustainable world. Activism can indeed be considered as a key requirement to get companies to change and to lead the way toward a more sustainable economy (Earl and Kimport, 2011). Our article brings support to this view, and probably even magnifies it, by showing that the firms that self-regulate are not only the ones that are criticized in activists’ attacks, but also those who observe others being attacked and decide to pre-empt. The real impact of activism is thus larger than one might believe by just considering the direct effect of attacks on targeted firms. On the other hand, our results also suggest that the development of more sustainable practices will have to come from other sources than private politics for certain firms, which will not respond positively to private politics attacks. In sum, the impact of private politics is thus larger than if one only considers the effect of direct attacks, but also not big enough to fully transform entire industries.

### Limitations and future research directions

This study also has limitations that create future research opportunities. First, our model focuses on environmental activism and EP. While the same model could be applied to social activism and social performance, there could also be some firms that use investments in environmental (social) measures to reduce the risks associated with social (environmental) activism in the future. For example, in the early 2000s, Walmart was mostly criticized for its labor practices, while the sustainability plan unveiled in 2005 was focused on the company’s environmental footprint (Plambeck and Denend, 2008). Future research could look into how this type of phenomenon could be integrated into our model of responsiveness.

There are also characteristics of our study that impose restrictions on the conclusions we may draw from our findings. One such limitation is that we assume that activists adopt a strategic approach to achieve maximum change within an industry, while in reality there is heterogeneity among activists (Krebs, 2001). This is unlikely to compromise the validity of our model as long as there are sufficient activists who strategically choose their targets to attract the public’s attention to the contested practice and companies believe that this is the case. However, future research should also look into whether managers take into account this heterogeneity of activists when they design their strategic responses to activism-related reputational risk. Moreover, while we focus on interactions between companies and activists, there are also interactions between activist groups and between industry peers. Future research should look into how these complex relationships can affect the real impact of private politics.

Our study also does not delve into the potential differences between the environmental issues for which an industry member is attacked and the types of practices its peers address after this attack. The reason is that our indicator of EP is an aggregate of a company’s practices related to the natural environment. Thus, it does not allow us to determine whether a company, once an activist is contesting an environmental practice, either addresses it, invests in environmental measures unrelated to this practice, or both. While we would expect that many companies ultimately address, at least in part, the industry practices that have been contested by activists, because it is important in terms of avoiding being the object of future attacks related to these practices, it would still be interesting to evaluate to what extent this is the case.

Our study is also focused solely on British companies. Since environmental and social performance is dependent on country-specific characteristics (Ioannou and Serafeim, 2012), company responsiveness to activist attacks with investments in EP could also depend on country-level characteristics, which future research should address.

Finally, it would be interesting to complement our study with some qualitative investigations within firms criticized by activists. This would be another way to evaluate whether managers do adopt a forward-looking perspective, but also to differentiate the different kinds of managers who might be involved in the decisions made. The general manager, for instance, might have a different stance on this than a public relations manager or a sustainability one.^5^ We hope scholars will consider this research avenue in the future.

## Conclusion

Most activist attacks seek to attract attention to industries’ questionable practices by strategically and publicly inflicting reputational damage on those industry members that will maximize the likelihood that the attack will garner media and public attention, in the hope that the industry will eventually change its practices. Taking this into account, this study integrates insights from the literature in private politics, corporate reputation, sustainability, and social movements to look into corporate responsiveness to the threat of activism within an industry. In this model, the driver of change is the ability of a firm’s proactive investments in self-regulation to reduce its future activism-related reputational risks, which is heterogeneous across firms in an industry and depends on the relative position of the firm in its industry regarding its existing EP. More work is certainly warranted, however, on the dynamic and future-oriented management of reputation within firms.

## Notes

1. In this, private politics differ from public politics, in which activist organizations or other interest groups “attempt to influence public office holders to the benefit of these groups themselves” (Baron, 2001), and thus mostly involve activities such as lobbying (Bonardi and Keim, 2005). Interestingly, private politics has become the preferred method of intervention for activists, over public politics: “Many activists have concluded that public politics is too easily blocked and have turned to private politics instead. Private politics refers to actions by private interests such as activists that target private agents, often in the institution of public sentiment” (Baron and Diermeier, 2007, p. 600).

2. In this article, we use the private politics definition of self-regulation. According to this literature, “Proactive measures by potential [activist] targets represent self-regulation, albeit self-regulation induced by the threat of strategic activism” (Baron and Diermeier, 2007, p. 61). Therefore, self-regulation is the adoption of environmental and social measures that go beyond what is required by regulation to prevent activist attacks or to reduce their damaging effects on the reputation of companies that have been, or could potentially be, targeted.

3. The reputation insurance literature also studies how investments in corporate social responsibility (CSR) can protect the company’s future reputation in general (Godfrey, 2005; Godfrey et al., 2010; Minor and Morgan, 2011), but it does not consider the relationship between firms and activists or how the former anticipate the risks associated with activist attacks.

4. We evaluated whether a company’s response to an activist attack on an industry peer, with an increase in environmental performance 2 years afterward, depends on the targeted company’s size or position in the industry’s distribution of environmental performance. The data show that the effect is present regardless of these two characteristics.

5. The authors would like to thank one of the reviewers for suggesting this point.
